# Correspondence

**Published:** 1914-04

**Authors:** Charles Haase

**Affiliations:** Buffalo, N. Y.


### CORRESPONDENCE.

*This department is intended for the presentation of news and views not coming within the scope of other departments, and particularly to afford opportunity for discussion and criticism of views elsewhere expressed.*

Buffalo, N. Y. March 14, 1914.

Dear Sir:

I have just returned from Berlin and Vienna where I spent six months studying. I must say that I have found a great change in Medical line in the two cities from what it was seven years ago. Vienna has practically stood still. They have finally finished two parts of the new hospital. The old general hospital is the same as it was years ago. At Berlin there has been great advancement made in post-graduate work, and it is now far superior to what one can get in Vienna. The cost of courses and living are much less in Berlin. Medical instruction in Berlin has not been commercialized as it has been in Vienna. In both cities they have good club rooms for American physicians.

Had a rather rough return voyage—twenty-three days on the Atlantic.

Yours truly,

CHARLES HAASE.

